# Circle of Vieussens: Its Importance in the Presence of Significant Coronary Artery Stenosis in a 26-Year-Old Female With Kawasaki Disease

**DOI:** 10.7759/cureus.66601

**Published:** 2024-08-10

**Authors:** Bilal Zia, Sajid Ali, Asif Ali, Abdalla Samman, Sami Farooqui, Emad Khalid, Shayan Khan

**Affiliations:** 1 Medical Affairs, Houston Cardiology Consultants, Houston, USA; 2 Cardiology, Houston Cardiology Consultants, Houston, USA; 3 Cardiovascular Medicine, UT Health Houston - McGovern Medical School, Houston, USA; 4 Internal Medicine, Cena Research Institute, Houston, USA; 5 Public Health, Cena Research Institute, Houston, USA; 6 Research, Cena Research Institute, Houston, USA

**Keywords:** kawasaki's disease, circle of vieussens in a patient with kawasaki's disease, circle of vieussens, chronic total occlusion percutaneous coronary intervention (cto pci), cardiac catheterization

## Abstract

In patients with coronary artery disease (CAD), collateral circulation aids in sustaining myocardial perfusion and cardiac function. The circle of Vieussens is a rare collateral pathway between the right coronary artery and the left anterior descending artery (LAD) that plays a significant role specifically in patients with chronic total occlusions (CTOs). This article presents a unique case of the circle of Vieussens in a 26-year-old Asian female with a history of Kawasaki disease and CTO of the proximal LAD. Despite the CTO, the patient remains asymptomatic and maintains normal left ventricular function, attributed to an effective collateral network including a right-to-left arterial ring providing TIMI 3 flow. The case illustrates the crucial role of collateral circulation in managing complex coronary anomalies and underscores the need for comprehensive cardiac evaluations in patients with Kawasaki disease. This finding also highlights the potential of the circle of Vieussens as a lifesaving alternate conduit in severe CAD scenarios.

## Introduction

In patients with total coronary artery occlusion, collateral circulation is crucial in maintaining myocardial perfusion and cardiac function. The conus artery, a small branch of the right coronary artery (RCA), often serves as a principal source of collateral circulation. In rare instances, a specific collateral channel known as the Circle of Vieussens, or Vieussens' ring, forms, providing a vital alternative blood supply route between the left anterior descending (LAD) artery and RCA [1]. This pathway is especially significant in patients with severe coronary artery disease (CAD), where well-developed collateral circulation can mitigate ischemic symptoms and preserve left ventricular (LV) function.

We describe a rare case of the circle of Vieussens in a 26-year-old Asian female with a history of Kawasaki disease. Kawasaki disease is characterized as vasculitis, often involving the coronary arteries, and can result in long-term cardiovascular complications; the condition is almost unheard of in adults [2]. The uniqueness of this case and its anatomical considerations make it noteworthy for clinicians managing patients with Kawasaki disease and complex coronary artery anomalies.

Informed consent was obtained from the patient for any materials used in the manuscript. This case report was conducted ethically in accordance with the World Medical Association Declaration of Helsinki.

## Case presentation

We present the case of a 26-year-old Asian female with a significant history of Kawasaki disease. She sought medical attention for left shoulder discomfort but denied experiencing shortness of breath, chest pain, or dizziness, whether at rest or during exertion. The patient's family history was devoid of cardiovascular diseases. Serum biochemistry was unremarkable, showing normal lipid levels, thyroid function, and electrolytes.

The cardiac evaluation included an unremarkable electrocardiogram (EKG) and echocardiogram, which showed normal LV function and no wall motion abnormalities. Coronary computed tomography angiography (CCTA) identified a possible dissection in the LAD, accompanied by high-grade stenosis at the junction between the first and second diagonal branches, necessitating a left heart catheterization to assess for epicardial stenosis and dissection.

**Figure 1:**
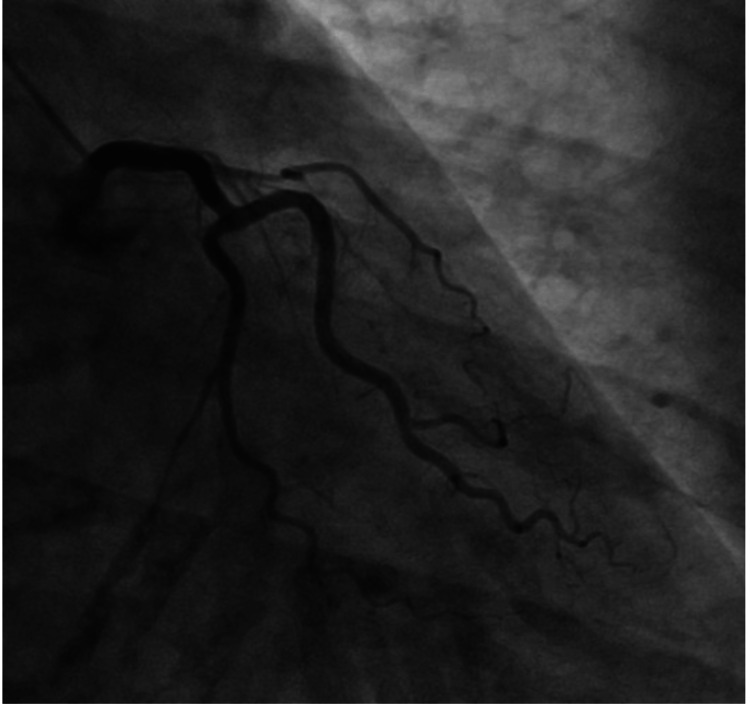
Left coronary angiogram Chronic total occlusion of the LAD in its proximal section LAD, left anterior descending artery

**Figure 2:**
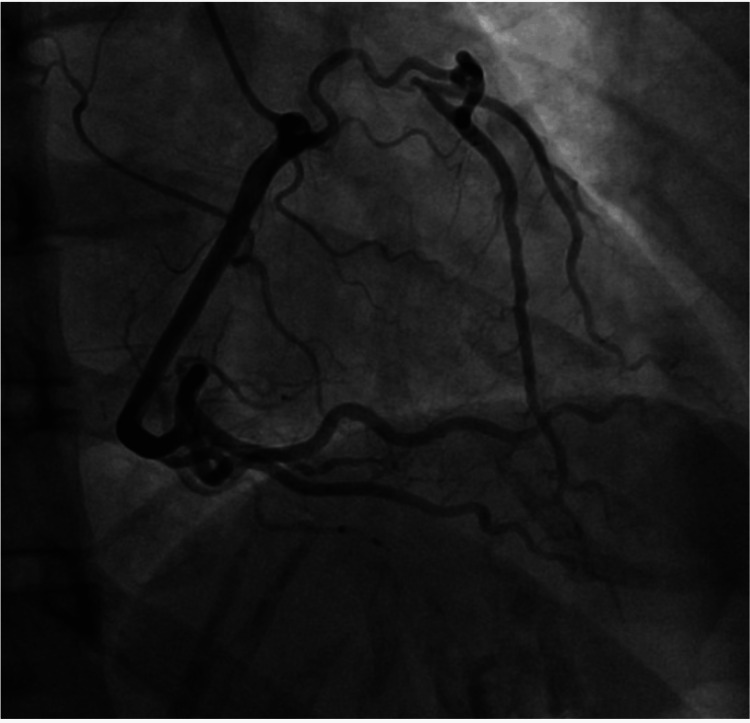
Right coronary angiogram A right-to-left 2-2.5mm collateral arterial ring can be seen with TIMI 3 ﬂow supplying blood to the LAD in an anterograde fashion from the RCA. LAD, left anterior descending artery; RCA, right coronary artery

Due to the unexplained shoulder discomfort and CCTA findings, a left heart catheterization was performed via left radial access using 5FR JR and JL4 catheters for selective coronary angiography (Figures 1, 2). The procedure unveiled a chronic total occlusion (CTO) of the proximal LAD just before a large diagonal branch. Both the left circumflex and RCA were clear of any disease. Notably, a right-to-left 2-2.5mm collateral arterial ring with TIMI 3 flow was observed, supplying blood anterogradely from the RCA to the LAD. This unique collateral configuration is known as the circle of Vieussens. Additional LV angiography of the aortic root confirmed an ejection fraction (EF) of 60%, with no signs of subclavian dissection or abnormalities in the aortic root. The patient was in stable condition and subsequently discharged.

## Discussion

Patients with CAD and CTO exhibit varying degrees of collateral circulation, significantly influencing ischemic symptoms and LV contractility. Collateral circulation can mitigate the effects of arterial blockages by providing alternative pathways for blood flow. The presence and extent of these collateral vessels are crucial in determining the clinical presentation and outcomes in patients with CAD and CTO [3].

The conus artery is a key component of coronary collateral circulation. However, it is not visible in approximately 20% of coronary angiograms, often because it arises independently or is missed due to distal contrast injection [4]. In around 30% to 50% of cases, the conus artery originates from an independent ostium in the right sinus of Valsalva, above and ahead of the RCA ostium, referred to as the isolated conus artery (ICA) [5,6]. This artery typically follows a short anterior and superior trajectory, supplying the pulmonary infundibulum and the supraventricular crest.

Occasionally, the conus artery forms an anastomosis with the LAD and the left conus artery, creating the arterial circle of Vieussens. This anatomical variation is critical in providing collateral circulation in the event of a proximal LAD occlusion. The arterial circle of Vieussens can significantly impact myocardial perfusion, especially when other major arteries are obstructed.

Collateral circulation generally contributes around 10% of the total myocardial blood supply. However, despite the proximal LAD CTO, our patient's 2-2.5mm-diameter circle of Vieussens provided adequate antegrade blood flow, keeping the patient asymptomatic with preserved LV function [7]. This highlights the importance of collateral pathways in maintaining myocardial viability and function in the presence of significant coronary artery stenosis.

The absence or occlusion of crucial collateral arteries, such as the circle of Vieussens, would likely result in severe ischemia in the LAD region, leading to adverse clinical outcomes. This demonstrates the necessity of identifying and preserving these collateral pathways while managing CAD and CTO.

In managing this patient's condition, we intend to continue with medical therapy and modify risk factors associated with Kawasaki disease, which can have long-term effects on coronary arteries. Kawasaki disease is known for causing coronary artery aneurysms and subsequent stenosis, making careful monitoring and management essential. Should ischemic symptoms arise, we plan to conduct a viability study using positron emission tomography (PET) scans to assess myocardial viability. Based on the results, we may consider minimally invasive revascularization options, such as using the left internal mammary artery (LIMA) to bypass the LAD.

## Conclusions

This case report underscores the significance of collateral circulation in patients with CAD, particularly those with CTO of major coronary arteries. Our presentation of a 26-year-old Asian female with a significant history of Kawasaki disease demonstrates how the circle of Vieussens can serve as a vital alternative blood supply route, preserving myocardial perfusion and LV function in the absence of direct arterial flow due to CTO of the LAD. Despite a complex clinical background and LAD obstruction, the patient maintained an asymptomatic status and normal LV function, attributed to the robust collateral circulation from the RCA through the circle of Vieussens.

This case highlights the clinical importance of recognizing and understanding collateral pathways, which can have profound implications for therapeutic strategies and patient outcomes. The detailed examination of this patient's collateral system provides insight into her unique anatomical adaptations while emphasizing the potential of collateral pathways such as the circle of Vieussens in managing similar cases of severe CAD.
